# Cataract
*An address to the Bristol Medico-Chirurgical Society on 13th February 1963.


**Published:** 1963-07

**Authors:** P. Jardine


### CATARACT*

BY

P. JARDINE

The subject on which I have elected to speak this evening was chosen for three reasons:—

(i) Cataract is one of the commonest causes of blindness or impaired vision in this country at the present time.
(ii) In a large proportion of cases the impairment of vision is remediable.
(iii) It is evident from my experience that some aspects of the condition are not generally as well understood as they might be.

The word itself is derived from the Greek and signifies "a pouring down", it being held by the ancients that the condition was due to an opaque humor from the brain running down behind the iris, thus forming a barrier between the pupil and the lens, the latter being considered to be the seat of vision and to occupy a central position in the eye. It is only some 250 years since the true nature of cataract, and indeed the real position of the lens was discovered and accepted by the scientific world, so it is hardly a cause for surprise if this knowledge has not penetrated to the lay public. In the West Country at least, patients still ask for the cataract to be taken "off" the eye.

At this point a few remarks on the development and functions of the lens might not be out of place. You will recall that at the beginning of the second month of foetal life the future lens can be recognized as a thickened area of the surface ectoderm overlying the optic vesicle, the outgrowth from the brain that will later form the retina. This ectodermal mass grows downwards, the stalk connecting it to the surface becomes attenuated and finally lost, and by the third month the cells have formed a hollow spherical mass, the lens vesicle, which secretes an elastic capsule on its outer surface. The cells of the posterior wall elongate to form lens fibres, thus obliterating the hollow of the vesicle, and constitute the primitive lens nucleus. The cells in the equatorial region continue to produce lens fibres throughout life, and thus the lens grows in size throughout life (a point of great practical importance), the oldest fibres being at the centre and the youngest at the periphery. The developing lens is nourished by a vascular layer which is derived from the hyaloid artery, but this is normally obliterated before birth, and thereafter the metabolic carriage is mediated by the aqueous humor. Specialized fibres of the vitreous attach themselves to the ciliary processes on the one hand and to the equator of the capsule on the other, thus forming the suspensory ligament of the lens.

The function of the lens of course is to focus the rays of light passing through it so that a sharp image is formed on the retina, but in this respect it is of secondary importance to the cornea, the refractive power of which is more than twice that of the lens. The lens, however, has the additional property of being able to increase its refractive power; this occurs when the ring-like ciliary muscle contracts during accommodation, relaxing the suspensory ligament, and so enabling the elastic capsule to mould the biconvex lens into a more nearly spherical shape. This ability is progressively lost with age, as the older, more central, fibres become more sclerosed. In old age the nucleus frequently develops a yellow or brownish tint, thus cutting off the passage of rays from the shorter (blue) end of the spectrum.

* An address to the Bristol Medico-Chirurgical Society on 13th February 1963.

### AETIOLOGY

By the term cataract we imply any opacity, in whole or in part, of the lens or its capsule. There are numerous varieties, which may be classified on an aetiological basis as follows:

### *Traumatic*

Cataract may result from concussion, or more commonly from a penetrating wound or intra-ocular foreign body, when damage to the capsule has been sustained.

### *Radiation*

Such cataracts were formerly found in certain industries where the workers were exposed to a heavy dosage of infra-red rays, as in steel chain-making and some forms of glass-making, but they are now rarely seen because the danger is known. Today they more commonly result from therapeutic radiation of structures in the vicinity of the eye.

### *Complicated*

This term is used when cataract occurs as the result of inflammation elsewhere in the eye, for example in cyclitis, and is probably the result of an altered composition of the aqueous.

### *Hereditary and Congenital*

Certain forms of cataract are known to occur in families, and are inherited in a dominant fashion, e.g. Coppock cataract; while others have a less regular mode of inheritance. Of the congenital forms the most important is that occurring when the mother has had rubella in the first trimester of pregnancy, and the earlier the infection the smaller and more central the opacity. Cataract can also occur in toxoplasmosis and galactosaemia.

### *Systemic and Toxic*

These forms occur in a wide variety of conditions—diabetes, mongolism, myotonic dystrophy, hypocalcaemia, associated with atopic dermatitis, and in poisoning by thallium and ergot. Iatrogenic cataract also followed the use of dinitrophenol, formerly used as a slimming agent. More recently cases have been reported due, it is thought, to steroids being given systemically in fairly high dosage over a long period.

### *Senile*

All the forms of cataract mentioned above are, with the exception of traumatic and congenital forms, comparatively rare, and although of interest to the specialist are of no great importance as a cause of blindness when compared with the senile form. Lens opacities can, if sought for, be found in the majority of people over middle age, and the incidence has been shown to follow what the statisticians call a saturation curve; that is, if we live long enough we get it, as it is part of the normal ageing process.

Briefly, it appears that the lens becomes more acid with age; the lens proteins tend to break down into more osmotically powerful particles and more water enters the lens (the intumescent stage), thus increasing its volume and reducing its clarity, until eventually the whole lens becomes opaque or "mature". At a later stage (hypermaturity) diffusion of the products of protein break-down may result in a shrunken lens, or if the capsule becomes impermeable a Morgagnian cataract may ensue, the nucleus lying free in a bag of protein-rich liquid.

Glaucoma due to angle-closure may develop during the intumescent stage, the swollen lens pushing the root of the iris forward. During the hypermature stage uveitis may be produced by the toxic effect of the protein break-down material, with or without an accompanying glaucoma. Also in the hypermature stage dislocation of the lens into the anterior chamber or into the vitreous may occur.

### DIAGNOSIS AND PROGNOSIS

Diagnosis usually presents no great problem. Where babies are concerned the practitioner may be consulted by the mother or nursing attendant who has noticed a grey or white appearance in one or both pupils. This only occurs when the opacity is anterior, or if central, fairly large. When the opacity is situated in the posterior part of the lens, or is small, it often escapes notice, and in these cases advice is not usually sought until later when, if the condition is binocular, it becomes obvious that the child's vision is not developing normally. In monocular cases the first sign may be that a squint develops—usually divergent—due to amblyopia; but some cases remain undetected until the child is sight-tested at a school medical examination.

In adults advice is sought because of visual symptoms. Most text-books state that the patient may complain of spots before the eyes. This symptom in my experience is rather rare, and what is usually complained of is a general dimness of vision (for which the patient's glasses—frequently new ones—are often blamed), or else a lowered acuity which may affect either distance or reading sight. Opacities in the posterior part of the lens have a greater effect on reading vision, and comments on the declining standard of modern newsprint are to be expected. When the cataract is accompanied by nuclear sclerosis it is the decline in distance sight (from the resulting myopia) that is chiefly noticed.

When the lens opacities are solely or mainly peripheral, the patient may notice that his sight is better in a bright light, when of course the pupil contracts and the light rays pass only through the clear, or relatively clear, central part of the lens. The opposite is true when the opacities are central; then the patient finds his handicap greatest in bright light when the pupil contracts, and quite appreciable improvement may occur in dim illumination when the pupil dilates. In chronic glaucoma under treatment with miotics even relatively slight lens opacities, if central, may call for glaucoma surgery so that the pupil may safely be allowed to assume its normal size. Lens opacities may also split the light into two or more beams, and so produce diplopia, or even polyopia, so that the patient sees two, three, or even more moons. This is one of the rare causes of monocular diplopia.

When the lens changes are confined to nuclear sclerosis and no actual opacities are present the medical practitioner is less likely to be consulted, the patient no doubt being of the opinion that the satisfaction of being able to throw away his reading glasses—which he often does—far outweighs the disadvantage of having to wear glasses to see the village clock or to read the hymn numbers in church, which he never had to do before; but he may have a poor opinion of his optician when glasses of increasing minus power become necessary for clear distance vision.

If the lens is completely or nearly opaque, or if in the case of congenital cataracts the opacity is either anterior or central and large, inspection with the naked eye using direct illumination may suffice to reach a diagnosis; but in all other cases the use of the retinoscope (merely a plane mirror with a hole in it) is called for. Retro-illumination, that is, the light being reflected from the fundus, will show up lens opacities as dark areas against the orange-red reflection from the choroid. One should remember that even in the complete absence of lens opacities the pupils of the elderly often appear slightly greyish owing to the increased reflection of light from the nucleus. The use of the retinoscope or ophthalmoscope will prevent erroneous interpretation of this.

For more detailed examination the ophthalmoscope is needed, using a lens of about +8 dioptres. Dilatation of the pupil with 1-2 per cent homatropine makes examination easier, and it is essential when there are peripheral opacities; but due caution should be exercised, bearing in mind that increased intra-ocular tension may exist, and that an attack of acute glaucoma may be precipitated by the mydriasis in subjects with shallow anterior chambers and narrow filtration angles which are common in the elderly. So it is better to avoid the use of atropine drops in such subjects. At the end of the examination it is advisable to abolish the mydriasis by the use of a miotic such as eserine drops ($\frac{1}{4}$ per cent).

No diagnosis of cataract should be made without an attempt being made to examine the fundus in order to exclude any co-existing pathological state such as the cupped discs of chronic glaucoma, senile macular degeneration, hypertensive retinopathy, and so on. When any doubt exists a specialist opinion is desirable, because a satisfactory view of the fundus may be impossible at a later stage when the lens opacities have progressed, and the decision as to whether or not to recommend surgical removal usually depends very largely on the health of the choroid, retina, and optic nerve. In even the most mature cataract the eye should be able to perceive light and have accurate projection, that is the patient should be able to point to the source of a light beam projected onto the eye. In the absence of accurate projection the prognosis should be cautious, as this state is usually indicative of additional retro-lental pathology —although cataract operation may still be desirable, and indeed necessary to reach a diagnosis. When no perception of light is present the prognosis for sight is hopeless.

A question frequently asked by the patient in whom cataract is diagnosed is how quickly will it develop to the stage when operation will become necessary? One should be rather cautious about committing oneself on this point. Congenital and hereditary cataracts are usually, but not always, non-progressive. Traumatic cataracts nearly always proceed quite quickly to maturity, but rarely the opacity due to a penetrating wound or foreign body remains localised. The rate at which the senile type increases varies greatly, but in general those occurring in younger patients tend to progress more quickly than those in the very aged.

In uni-ocular cataract the limited benefits of operation will have to be explained to the patient. When the fellow eye is emmetropic, the provision of a spectacle lens for the treated eye results in a retinal image which is about one-third larger than that in the untreated eye, and owing to this disparity the brain is unable to fuse the two images, so that diplopia is experienced. For this reason special measures, which I shall deal with later, are required; but even without these it may be desirable to remove a uni-ocular cataract if it is causing trouble because of hyper-maturity or for any other reason, or if the restoration of a full visual field is desirable because of occupational or other special hazards. We are so accustomed to assessing visual capacity in terms of central visual acuity that we sometimes overlook the importance of peripheral vision.

When a diagnosis of cataract has been reached, the question arises as to what the patient should be told. In general it is best to tell the truth—lest someone else do so! But the very word cataract has such sinister connotations for so many patients, conjuring up visions of total blindness, that when this fear is known to exist it may be better to use some euphemism such as "lenticular opacities", or "lens haze", to explain their common occurrence in the later decades, and to given an encouraging forecast of the results of operation should this ever become necessary.

### TREATMENT

Treatment of cataract may be called for:—

(i) To improve or restore vision, or to restore binocular vision in cases of uniocular cataract,

(ii) To see the fundus clearly for diagnostic purposes, as when an intra-ocular neoplasm is suspected, or to facilitate other surgical procedures, such as the treatment of retinal detachment,

(iii) For the relief of uveitis or glaucoma due to hypermaturity of the lens, or of glaucoma resulting from intumescence of the lens,

(iv) For cosmetic reasons, especially in patients with dark irides.

Of these indications the commonest is the need to improve the patient's vision. General experience has shown that when the acuity is less than about 6/18 operation is most often called for, but no hard and fast rule can be laid down. The decision really rests on the extent to which the patient's activities, either of work or leisure, are being curtailed. The deciding factor in one patient may be inability to read a car number plate at 25 yards (which means approximately 6/12 vision), in another failure to read the hymn numbers in church, and in a third difficulty in seeing the share prices in the Financial Times.

Since removal of the lens also means the abolition of the power of accommodation I do not recommend surgery in young people unless acuity is less than 6/24 or even 6/36; and there is a body of well-informed opinion which holds that it is wiser to operate on one eye only because of the possibility that retinal detachment may occur 15 to 25 years later. But in babies, if the extent of bilateral opacities makes it obvious that operation will ultimately be required, the earlier this can be done the better, in order to safeguard the proper development of macular fixation and to prevent nystagmus; but the technical difficulties of cataract operations in infants of less than one year are notorious.

What means of improving vision are at our disposal? First let it be said that apart from the rare occasions when opacities are due to oedema and are reversible, as in diabetes and galactosaemia, medical treatment is of no avail, although it has been attempted in many forms, varying from the honestly held belief in the efficacy of potassium iodide, vitamins, or diet, through the pure ju-ju of injection of fish-lens protein, to the charlatanism of exposure to coloured lights.

Palliative treatment may afford some measure of benefit. When the opacities are mainly peripheral, a good bright light in a suitable position, say from an "anglepoise" type of lamp, may be of help; or the pupils may be contracted with a weak miotic such as $\frac{1}{2}$ to 1 per cent pilocarpine. When the opacities are central, dilatation of the pupils should be encouraged by using a rather weak illumination for reading, or by using a simple dark mask over the page, with a narrow aperture cut in it to expose a line or two of print at a time. Such a mask can be made quite easily, or purchased, and can be obtained with an adjustable aperture. Out of doors a wide-brimmed hat or tinted glasses may help, and mydriatics may be used—homatropine rather than atropine in the elderly. In the young, a mydriatic such as phenylephrine (10 per cent), which does not abolish accommodation, may be useful.

When palliative measures fail to help, surgery is the only alternative. With central non-progressive opacities which are not too large (that is when full mydriasis yields a satisfactory improvement in sight) an optical iridectomy may be considered. This entails removing a segment of iris, usually in the lower nasal quadrant, so that light may pass through the clear part of the lens. But usually in patients who benefit from this procedure just as much help can be obtained from wide mydriasis, though iridectomy relieves the patient from the tiresome necessity of the constant use of drops. The other and much more common surgical treatment is removal of the lens.

This may be accomplished in children and young adults (say up to the age of 25 or 30) by the operation of discission or needling, in which a "needle" (actually a very narrow-bladed knife) is passed through the cornea near its limbus and an incision is made in the lens capsule, so that the aqueous gains access to the lens fibres, which then swell, break up, and dissolve. The operation may have to be repeated on several occasions, and it is essential that the pupil be kept dilated until the lens-matter is completely absorbed; otherwise adhesions between the iris and the lens-capsule will form, and prevent dilatation of the pupil and handicap further attempts at needling. An acute rise in intra-ocular pressure may follow needling. This may be due either to swelling of the lens which pushes the iris forward so that the filtration angle is blocked, or to blockage by particles of soft lens-matter. Formerly this complication called for a paracentesis of the cornea, and the removal by irrigation of as much of the soft lens-matter as possible; but with the introduction of carbonic anhydrase inhibitors (acetazolamide or "Diamox") it is frequently possible to keep the pressure under control until with continuing absorption of the lens-matter the cause of the complication is removed.

Needling can only be used in young subjects with no hard nucleus in the lens, when the central and oldest part of the lens has not yet become sufficiently hard to resist hydrolysis. No fixed age can be laid down for this, but is generally regarded as somewhere about 25 or 30.

In older subjects the removal of cataract entails opening the eye. In ancient times "couching" of the cataract, that is, depressing it into the vitreous, is said to have been practised, and still is to this day in the more remote parts of India. After an initial improvement in vision, the eyes in these cases are generally lost, either because of infection at the time of operation, or because endophthalmitis results from the allergic response of the eye to the lens-matter.

Credit for the first operation in historical times is given to a Frenchman, Daviel, who performed it in 1748, but the method did not become generally practised until about 100 years later. At first, all that was done was to make a corneal incision, open the lens capsule with a knife or tear away part of it with toothed forceps, and express the lens or at least its nucleus. This is the extra-capsular operation. If much soft cortical lens matter remained behind severe post-operative iritis resulted. The less soft lens-matter remaining, the better were the results, and this is why cataracts were left until they became fully "ripe". Other common and important post-operative complications were prolapse of the iris through the corneal wound, and gaping of the wound, both liable to lead to infection and loss of the eye. Prolapse could be prevented by removing a complete segment of iris near the incision, and gaping could be minimized by continuing the corneal incision under the conjunctiva, to form a flap which soon adhered to the episcleral tissues and led to quicker healing. Later it was found that a peripheral or basal iridectomy prevented prolapse, and had the advantage of being cosmetically more attractive. Since the cutting of the iris was generally the painful part of the proceeding for the patient (the operation being conducted under indifferent topical anaesthesia with cocaine drops) this was often performed as a separate operation several weeks before the cataract extraction.

Post-operatively the patient had to remain quietly at rest in bed for at least a week, taking care to avoid sneezing, coughing, or squeezing the eyes, since rupture of the new vessels growing into the conjunctival flap was liable to occur, and a hyphaema to result. The hands were usually tied down to the sides of the bed at night to prevent accidental rubbing of the eyes during sleep. Following the cataract extraction it was often necessary to make an opening in the remaining posterior capsule if this was thick or wrinkled, and this was especially likely to be necessary if any substantial amount of soft lens-matter had been left behind.

In the twenties of the present century the intra-ocular method, whereby the capsule is grasped with non-toothed forceps and the lens removed intact, began to find acceptance. About the same time local infiltration anaesthesia and akinesia by injection of procaine solution into the orbit and facial nerve gained in popularity. Later still, with the improvement in suture materials and needles, suturing of the incision with or without a conjunctival flap became more common, as did the practice of injecting an air bubble into the anterior chamber at the conclusion of the operation to act as a buffer and absorb any rise in intra-ocular pressure caused by coughing or squeezing the eyes. This air is absorbed in a few days.

The next important advance was the introduction of alpha-chymo-trypsin. This proteolytic enzyme dissolves the suspensory ligament of the lens (zonulolysis), thus facilitating extraction by the intracapsular method, especially in young subjects in whom the ligament tends to be stronger than the capsule. At the same time an improved version of the erisiphake became very popular, in which the lens is held by suction—an old idea revived—especially in combination with zonulolysis; and the idea was introduced of temporarily suturing a wire ring to the sclera to prevent its collapse when the vitreous was known or suspected to be fluid and thus liable to prolapse.

This probably represents a fair picture of cataract surgery as it stands today, but it must be emphasised that there are as many modifications and opinions as there are ophthalmic surgeons. Some consider local anaesthesia out-moded and insist on a general anaesthetic. Some believe that the extra-capsular operation is safer, on the grounds that it is less likely to lead to retinal detachment. Others prefer a complete to a peripheral iridectomy as it makes removal of the lens easier, and if a retinal detachment does ensue it is easier to see it and treat it successfully.

The advances in surgical treatment have led not only to a higher percentage of good visual results, but also, and more important, they have, by allowing the patient to get up as early as the second or third post-operative day, reduced the incidence of complications such as thrombosis and pulmonary embolism once so dreaded in elderly subjects confined to strict bed-rest.

After cataract removal the eye is hypermetropic if formerly emmetropic, more so if formerly hypermetropic, and less so if formerly myopic. An eye formerly myopic to the extent of 24 dioptres requires no distance correction at all after cataract removal. The uncorrected visual acuity of a formerly emmetropic eye is about 3/60 when the cataract is removed, so that even without correction the patient can manage to get about, but all images are blurred and enlarged—he sees "men as trees walking"—and glasses of about +10 dioptres are needed for clear vision. These are not prescribed until some seven or eight weeks after operation, as the shape of the cornea changes slightly during this time. Although these glasses may give a visual acuity of 6/6 or even better, they have certain disadvantages. They are thick, heavy, and not very sightly, and even if these objections can be partly overcome by the use of reduced-aperture lenses or by plastic lenses, other optical disadvantages remain, of which the most important is spherical aberration, a doorway for example appearing barrel-shaped. There is also the prismatic effect of the periphery of the spectacle lens, which necessitates the use of its central portion only, and this means moving the head, not the eyes, when looking at objects to the side. This is particularly a disadvantage to car drivers attempting to reverse.

In uni-ocular aphakics, owing to the disparity in size of the images in the treated and the untreated eye, correction of the aphakia by spectacles is ruled out. In order to overcome this disparity the idea of substituting an intra-ocular artificial lens was introduced by Ridley some twelve years ago. This is a lens made of plastic material which is placed in the position of the natural lens immediately after extraction of the cataract. Many successes were reported, but there was a high proportion of failures as well, due either to uveitis or to the lens becoming subsequently dislocated into the vitreous. A later development was the anterior chamber implant of Strampelli, which at first looked like being the answer to the problem. However, a certain number of these have given rise to corneal opacity after having been apparently satisfactory in every way for several years, so that a conservative attitude is best adopted towards these methods.

The other method of overcoming the disparity in size of the retinal images lies in the use of a contact lens. By this means the inequality can be reduced to a level (5–10 per cent) at which the brain is able to fuse the two images so that binocular vision can be achieved. The success of this depends largely on the skill with which lenses are made and fitted, and on the ability of the patient to tolerate them and to learn to insert them; and this is a trick more easily acquired by the young than by the more elderly, as may be well understood.

